# When Lying Feels the Right Thing to Do

**DOI:** 10.3389/fpsyg.2016.00734

**Published:** 2016-06-02

**Authors:** Sophie Van Der Zee, Ross Anderson, Ronald Poppe

**Affiliations:** ^1^Computer Laboratory, University of CambridgeCambridge, UK; ^2^Department of Medical and Sport Sciences, University of CumbriaLancaster, UK; ^3^Networked Organisations, TNORijswijk, Netherlands; ^4^Department of Information and Computing Sciences, Utrecht UniversityUtrecht, Netherlands

**Keywords:** deception, dishonesty, rejection, insurance fraud, MTurk

## Abstract

Fraud is a pervasive and challenging problem that costs society large amounts of money. By no means all fraud is committed by ‘professional criminals’: much is done by ordinary people who indulge in small-scale opportunistic deception. In this paper, we set out to investigate when people behave dishonestly, for example by committing fraud, in an online context. We conducted three studies to investigate how the rejection of one’s efforts, operationalized in different ways, affected the amount of cheating and information falsification. Study 1 demonstrated that people behave more dishonestly when rejected. Studies 2 and 3 were conducted in order to disentangle the confounding factors of the nature of the rejection and the financial rewards that are usually associated with dishonest behavior. It was demonstrated that rejection in general, rather than the nature of a rejection, caused people to behave more dishonestly. When a rejection was based on subjective grounds, dishonest behavior increased with approximately 10%, but this difference was not statistically significant. We subsequently measured whether dishonesty was driven by the financial loss associated with rejection, or emotional factors such as a desire for revenge. We found that rejected participants were just as dishonest when their cheating did not led to financial gain. However, they felt stronger emotions when there was no money involved. This seems to suggest that upon rejection, emotional involvement, especially a reduction in happiness, drives dishonest behavior more strongly than a rational cost-benefit analysis. These results indicate that rejection causes people to behave more dishonestly, specifically in online settings. Firms wishing to deter customers and employees from committing fraud may therefore benefit from transparency and clear policy guidelines, discouraging people to submit claims that are likely to be rejected.

## Introduction

Fraud is a pervasive and expensive problem: estimates of the cost of fraud vary from under 1% of GDP to over 10%, with the largest recent estimate of global fraud costs put at £7.22 trillion, or one seventh of global GDP (Gee and Button, 2013). A more modest estimate suggests that fraud is costing UK citizens approximately £1100 a year each (Centre for Counter Fraud Studies, 2014). According to the UK Fraud Act, fraud consists of “dishonestly making a false representation with the intent to make a gain for oneself or another, or to cause loss to someone else”: in short, “an act of deception intended for personal gain or to cause a loss to another party,” as the Serious Fraud Office summarizes the law.

In this paper, we focus on deception in online settings. A detailed study suggests that, while ‘technical’ offenses such as payment card fraud, online banking fraud, and Internet fraud have an annual cost of several 10s of pounds per citizen per year, online versions of traditional offenses such as tax and welfare fraud costs each citizen of a developed country 100s of pounds a year (Anderson et al., 2012). And these are just the financial costs. When the fraud victims are persons rather than institutions, they can also experience negative psychological consequences, increased physical and mental health issues and damage to their relationships (Button et al., 2014). Motivated by the high direct and indirect costs of fraud, a wide range of countermeasures have been introduced (Alanezi and Brooks, 2014; Centre for Counter Fraud Studies, 2014). These include increasing security and surveillance, increasing awareness amongst potential victims and calling for more vigorous prosecution of fraudsters (Anderson et al., 2012; Purkait, 2012); they unfortunately also include measures such as blaming fraud victims for their misfortune (Cross, 2013). Although these measures may be rational from the viewpoint of the actors who introduce them, they do not always take the more irrational side of human nature into account.

Not all fraud is committed by ‘professional criminals,’ that is by individuals who earn their living through committing offenses. Instead, both real-world case studies and experimental research have shown that very few people lie and cheat to a pathological extent (for an overview of experiments, see Ariely, 2012); instead the majority of people are ‘opportunistic fraudsters’ who lie and cheat a little. Padded expenses, inflated insurance claims, refunds for goods wrongly said to have been defective, overtime payments for tea breaks; the world of trade, commerce and employment are beset with dishonest behavior. In a series of experiments involving participants who tried to solve as many matrix puzzles as they could within several minutes (Mazar et al., 2008; Gino et al., 2009), it was consistently found that people overstate their achievements by about 60% if they have the chance.

To prevent people from deceiving, we need to understand the factors that cause people to behave dishonestly. The deterrence of deception lies at the heart of most fraud countermeasures. In the last decade, vibrant research on the deterrence of deceit and dishonesty has emerged. This paper is aimed at a better understanding of when dishonest behavior occurs. Previous research has demonstrated that the extent to which people behave dishonestly is affected by several factors including individual differences, context and the environment. Creativity is one example of an individual difference that is linked to dishonesty. Creative people are more likely to behave dishonestly (Gino and Ariely, 2012), and are also better at it (Vrij, 2008). While individual differences can cause some people to be more dishonest than others, situational factors can increase the likelihood still further. Dishonesty tends to fluctuate during the day (Kouchaki and Smith, 2014). Throughout the day, people become more depleted, lowering their moral awareness and self-control. Therefore, dishonesty tends to increase as the day progresses. People tend to be more dishonest under certain circumstances, for example when pursuing a goal (Schweitzer et al., 2004), or in the presence of a bad example such as counterfeit goods (Gino et al., 2010). The behavior of other people can also influence a person’s tendency to act dishonestly. For example, people are more likely to behave dishonestly when witnessing an in-group member behaving dishonestly (Gino et al., 2009), and when feeling socially rejected (Kouchaki and Wareham, 2015). The latter effect was mediated by physiological arousal, a finding that is in line with previous research suggesting that feelings of anxiety can promote dishonesty (Kouchaki and Desai, 2014). Therefore, non-social situations that elicit feelings of anxiety may also elicit dishonest behavior.

These situational factors have in common that they can be used to justify unethical behavior. Blasi (1980) identified a psychological gap that can emerge when people’s moral understanding and their moral actions are not aligned. A possible explanation for the mental processes that go on when this misalignment happens is the occurrence of ethical dissonance. Ethical dissonance can be triggered by the desire to uphold a positive moral self-image, and the temptation and potential benefits associated with unethical behavior (Barkan et al., 2015). The theory describes a conflict between two opposing factors: on the one hand, people want to benefit as much as they can and dishonesty may increase their benefits (Mazar et al., 2008), while on the other hand they want to view themselves as good and honest people (Aronson, 1969; Josephson Institute of Ethics, 2012). Behaving dishonestly may threaten their positive self-concept, but this threat is mediated by the justification of this immoral behavior. These justifications may occur both before (i.e., anticipated ethical dissonance) and after unethical behavior (i.e., experiences ethical dissonance; Shalvi et al., 2015). The empirical evidence is that people are much more prepared to cheat when the extra amount of money or working time is relatively small or can otherwise be rationalized (Ariely, 2012).

Previous research has indicated that social rejection, and the anxiety associated with this rejection, can lead to increased dishonest behavior (Kouchaki and Wareham, 2015). However, not all rejections are social in nature, and the effect of other types of rejection on dishonest behavior remains unclear. In this paper, we focus on the situation in which people’s efforts are rejected. We investigate different aspects of the rejection. Specifically, we look into the subjectivity of the rejection and the monetary reward associated with the rejection.

So far, most research on factors that induce dishonest behavior was carried out in the lab. Although lab studies benefit from high experimental control, it remains unclear how findings obtained in a lab translate to an online setting. In a world where technological developments have enabled people to increasingly perform a variety of activities online, it is important to understand how an online context affects people’s behavior. Previous dishonesty research has indicated that people may behave differently when they act online. For example, in 15-min long conversations, participants lied more often during online conversations compared to face-to-face interactions (Zimbler and Feldman, 2011). Therefore, we investigate whether the rejection of one’s efforts also increases dishonesty in an online setting. This may not only apply to dishonest behavior in general, but also specifically to the effect of the nature of a rejection on dishonest behavior.

## General Materials and Methods

This paper contains three studies that were reviewed and approved by the Research Ethics Committee of Cambridge University’s Computer Laboratory. The studies were conducted online. Each experiment started with an information sheet in which participants were informed that they were about to participate in an academic survey (Study 1) or study (Studies 2 and 3). In Study 1, participants were told that the survey was designed to test the language proficiency of the American population. The survey consisted of some general questions and two language related tasks, one grammar and one semantic task. For Studies 2 and 3, participants were told that they were participating in a study to test a newly developed Automatic Validation Tool and that the study involved answering some general questions and filing a mock insurance claim. At this stage, participants were not informed about the true nature of the study, measuring dishonest behavior, because this knowledge could influence their behavior. It was explained that the study would start when clicking “next,” and that by doing so they gave their consent to participating in the academic survey/study. At the end of each study, participants were debriefed in writing about the true purpose of the study, and we explained why we could not reveal the deceptive nature of the research earlier. We also asked participants not to share the true nature of the study online until data collection was finished in order to avoid data pollution. As part of the debriefing, all participants were offered the opportunity to contact the experimenters with any questions or complaints, or to retract their data.

The experiments were conducted on Amazon’s Mechanical Turk (MTurk), an online platform that is frequently used to collect experimental research data. This recruitment channel was deliberately chosen for two reasons. First, studying dishonest behavior in an anonymous, online environment extends the existing dishonesty literature. Second, experimental research has shown that recruiting on MTurk leads to a representative sample of the U.S. population (Berinsky et al., 2012). This is a more varied participant sample than we would have been able to gather at our university and the variety increases the generalizability of the presented findings within the American population. Another benefit of conducting experimental research on MTurk is the low cost compared to lab experiments, without loss of validity. That low pay does not influence the quality and nature of research results was demonstrated by Paolacci et al. (2010), who replicated a series of classic decision-making studies using MTurk and found similar results to the more expensive original studies that were collected in the lab. Similarly, in Ariely’s (2012) experiments, increased financial incentives did not lead to an increase in cheating.

We have studied the effect of different types of rejection (objective, subjective, with promised financial reward, and without) on ethical decision-making using two different types of experimental research designs. We purposefully designed experimental procedures that resembled real-life situations in which the occurrence of dishonest behavior is prevalent. Study 1 comprises a language proficiency study, in which we measured cheating behavior under truly experienced circumstances, while Studies 2 and 3 were both vignette studies in an online insurance claim context that involved participants responding to a hypothetical rather than experienced scenario. Vignette studies have been conducted in a wide range of disciplines including teaching (Poulou, 2001) and nursing (Hughes and Huby, 2002), and have proven particularly useful when studying sensitive topics such as violence in residential care homes (Barter et al., 2004), HIV risk in drug users (Hughes, 1998) and deception (Schweitzer et al., 2004). Due to the sensitive nature of dishonest behavior and insurance fraud, vignettes are a suitable research method for this topic. Although reading a vignette will likely differ from real-world experiences, experimental research has demonstrated that vignettes can provide a sufficiently realistic scenario to affect people’s responses (Hughes, 1998; Barter et al., 2004). We additionally added a cover story about testing of our newly developed Automatic Validation Tool to the vignette study to increase the plausibility of our request.

Anxiety, for example when elicited by social rejection, has been shown to affect dishonest behavior (Kouchaki and Desai, 2014; Kouchaki and Wareham, 2015). Because anxiety may also play a mediating role in our rejection manipulation, we invited participants in all three studies to self-report how they felt before and after our manipulation. This allowed for measuring how participants were affected by own rejection manipulation, and whether the elicited emotions mediated the effect of rejection on dishonesty. Additionally, in the first study participants also reported how they felt after the cheating opportunity, to measure if cheating affected how people feel. Dishonesty was measured dichotomously based on actual cheating (Study 1) and lying (Studies 2 and 3) behavior.

## Study 1

### Methods and Materials

#### Participants and Design

One hundred and sixty-nine American MTurk workers participated in an online study on the effect of unfair rejection on people’s mood and cheating behavior. Although the majority of MTurk data is of high quality, some MTurk participants provide random answers. In order to identify these data polluters, we identified several *check questions* in each study. In Study 1, participants had to answer all 10 grammar questions correctly. This conservative criterion was required to operationalize the rejection of effort, see Section “Procedure” in Study 1. Thirteen people failed to answer the 10 questions correctly and were removed from the dataset, leaving 156 participants (94 female; age 18–79, *M* = 33.85, *SD* = 13.01). Of these, three participants did not have English as their native language. Participation took on average 12 min and participants received $1.70 for their time, consisting of a basic payment of $0.50 and a $1.20 bonus that each participant eventually received.

This study is a between-subjects design, measuring the effect of unfair rejection on cheating (cheating vs. no cheating).

#### Procedure

Participants accessed our website via the MTurk platform and were told that it was a study of English language proficiency, consisting of a grammar test and a semantics test. More specifically, the study was framed as a state-dependent retrieval study (i.e., the memory phenomenon that retrieval performance is affected by the mood and state during which the memories were initially formed; Eich, 1995), in which the effect of mood on test performance would be measured. State-dependent learning was the cover for asking participants to report their feelings regarding five different emotions on a 7-point Likert scale (i.e., happy, sad, guilty, frustrated, and anxious) three times: before and after the feedback (accept or rejection) and after the cheating opportunity. The first two mood questionnaires served a dual purpose. First, they served as a manipulation check, measuring the effect of rejection on participants’ feelings. The self-reported emotion ratings serve as a proxy for the perception of the treatment. The second purpose of the first two mood questionnaires was to identify whether the experienced emotions mediate the effect between rejection and cheating. The third questionnaire was included to measure whether the act of cheating affected people’s feelings.

The study started with demographic questions, and was subsequently divided into two parts: the grammar and semantics tests. Participants were told that they would receive a $0.60 bonus if they answered all 10 multiple-choice grammar questions correctly. The grammar questions served as a conservative check, and to ensure that participants had invested time and effort before their efforts were rejected. The 10 grammar questions also made our cover story of a language proficiency test more plausible. When participants failed to answer the 10 questions correctly, they received feedback that they would not receive the bonus. As this setting was not a planned manipulation, we excluded these trials from the analysis. Of the participants that answered all 10 questions correctly, half were provided with false feedback that they had answered the final question incorrectly (i.e., rejection condition). The other half did not receive such feedback and were told that they would receive the bonus for this part of the study (i.e., accept condition). Subsequently, participants were asked to provide the definitions of three words. For each correct definition, participants were promised $0.20, with a total of $0.60 if all three definitions were correct. The three words were chosen based on the results of a pilot study in which we investigated what words people do and do not know. Forty-seven were tested, and four participants were removed because they failed to answer the check questions correctly, leaving 43 participants (15 female; age 18–68, *M* = 37.21, *SD* = 14.48). We intended to include two words that all people know, and one word that no one knows. The pilot results indicated that people are familiar with the words ‘goal’ and ‘employee,’ but not with the word ‘kench.’ A kench is a deep bin to salt fish and animal skins, used by fishermen and sailors in the 18 hundreds. Today the word is obsolete and is unknown to the general population.

During Study 1, it was explicitly stated on the website that participants were taking part in a language proficiency test, and that looking up the correct answer was not allowed. Therefore, cheating was defined as providing the correct answer to the ‘unknown’ target word kench. Twenty-eight participants cheated by providing the correct definition of kench. We also observed another source of unethical behavior, when people quit the experiment with the presumed intention to start over to avoid missing out on the bonus. We had purposefully designed the website such that the back button was disabled and people could only participate from the same IP-address once. Consequently, participants did not succeed when attempting to access the experiment website for a second time. These measures were explicitly explained to all participants and were taken in order to avoid people going back to the previous page to change their answer after receiving the feedback. In total, 13 participants quit after receiving the feedback, and all quitting participants were part of the rejection condition. While participants could quit for other reasons, several participants emailed the experimenter to indicate their intention to start over the study in order to obtain the bonus. Excluding these participants from the dataset would provide a skewed view because it concerns meaningful rather than randomly missing data. Instead of omitting these 13 quitters from the analysis or treating them as cheaters, we consider Behaving Unethically as the broad class of dishonest behaviors, including cheating and quitting. In total, 41 people behaved unethically. After the cheating opportunity, participants were debriefed about the true purpose of the study. All participants received the full bonus of $1.20, regardless of performance and previous feedback. This decision was made in consultation with our ethics committee. This way, all participants were treated equally as payment was not dependent on experimental condition. Because participants were not made aware of this until the data collection was finished, it should not have affected the results.

### Results and Discussion

**FIGURE 1:**
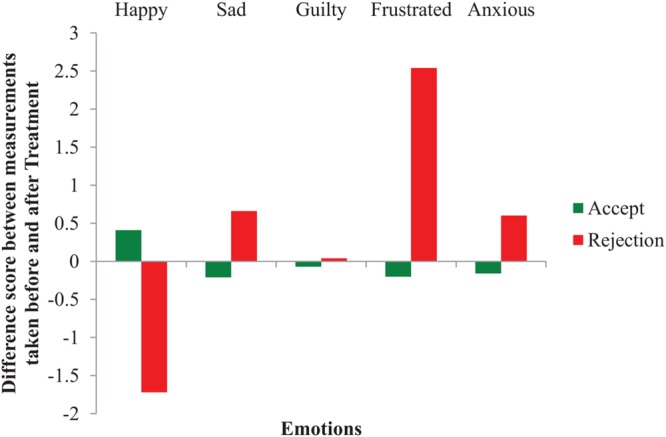
**The effect of Treatment on self-reported Mood (Study 1)**.

**FIGURE 2:**
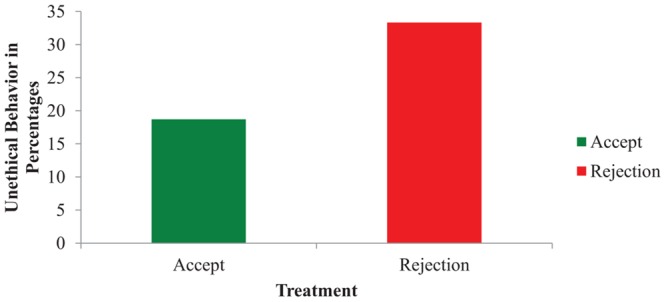
**The effect of Treatment on Unethical behavior (Study 1)**.

To measure whether our rejection affected people’s mood, participants were invited three times to indicate on a ‘not at all’ (1) to ‘very much’ (7) Likert scale how happy, sad, anxious, guilty, and frustrated they felt: before (time 1) and after (time 2) the feedback, and after the subsequent cheating opportunity (time 3). Five repeated-measures ANOVAs with Treatment (accept vs. reject) as the independent variable and five self-reported mood measures on times 1 and 2 as the dependent variables revealed four interaction effects, indicating our rejection manipulation was successful. Specifically, participants reported: (i) feeling less happy after being rejected, *F*(1,147) = 109.28, *p* < 0.001, $\eta_{p}^{2}$ = 0.43; but, (ii) more sad when rejected, *F*(1,147) = 23.69, *p* < 0.001, $\eta_{p}^{2}$ = 0.14; (iii) more frustrated, *F*(1,147) = 94.67, *p* < 0.001, $\eta_{p}^{2}$ = 0.39; and, (iv) more anxious, *F*(1,147) = 12.94, *p* < 0.001, $\eta_{p}^{2}$ = 0.08. However, guilt was not affected; see **Figure 1**, for a graphical interpretation of the results. Overall, these self-reported mood results indicate that participants’ mood was negatively affected by the rejection. To measure whether rejection also promoted unethical behavior, a chi-square analysis of Treatment on Unethical Behavior was performed. As demonstrated in **Figure 2**, people behave more unethically after rejection (33.3%) compared to being accepted (18.7%), *X*^2^(2) = 4.32, *n* = 156, *p* = 0.046, Φ = -0.17. The difference between these two conditions was predominantly caused by the participants that quit the experiment. In the accept condition, 14 out of 75 participants cheated (i.e., 61 participants did not cheat). In the unfair rejection condition, 27 out of 81 participants behaved unethically (i.e., 54 participants did not), of which 14 participants cheated by providing the correct definition of kench and 13 quit early. Because the latter group did not complete the mood questionnaires, it was not possible to run a mediation analysis to determine whether mood mediated the effect between rejection and dishonesty. Finally, to measure whether behaving unethically affected people’s emotions, a MANOVA was performed of Unethical Behavior and Treatment on five self-reported mood measures on time 3. This test revealed that people’s emotions after the cheating opportunity were affected by Treatment, *F*(5,136) = 6.89, *p* < 0.001, $\eta_{p}^{2}$ = 0.20, but not by Unethical Behavior, *F*(5,136) = 1.32, *p* = 0.260, $\eta_{p}^{2}$ = 0.05.

A first interesting finding is that cheating in itself did not cause an emotional response. One of the main theories of the detection of deceit is based on the assumption that lying can cause an emotional response (Zuckerman et al., 1981; Vrij, 2008). One hypothetical explanation for this discrepancy between the literature and our finding is that in our study participants cheated (looked up a word while this was not allowed) rather than lied (provide falsified information). Although lying and cheating are both dishonest behaviors, they may elicit different responses. Alternatively, dishonest behavior in general does not always cause an emotional response, for example when there is little at stake. In our study, there were no clear negative consequences to getting caught cheating. If dishonesty does not necessarily elicit an emotion response, this would have consequences for the generalizability of the emotional approach as the base for a lie detection method. This hypothesis has found some support in the deception community, which has shifted from an emotion-based lie detection approach to a cognitive load-based approach over the last decade (Vrij et al., 2015). Future research is needed to investigate the emotional response to different types of dishonest acts in order to determine the generalizability of an emotion-based lie detection approach.

The second interesting finding is that rejection caused both more negative emotions and more dishonest behavior in this online cheating environment. We operationalized rejection by unfairly rejecting the participants’ correct answers and thereby taking away their financial reward. That this rejection was perceived as a negative experience was demonstrated by the self-reported mood data; rejection caused people to feel sad, anxious, frustrated, and unhappy. However, based on the current data we cannot determine whether the negative emotional response mediated the effect between rejection and cheating. Therefore, for Study 2, we implemented a new research design in which participants could not cheat by quitting halfway through the experiment. In addition, participants in the current study were rejected on unfair grounds (i.e., although participants answered all questions correctly, they were told they made a mistake and therefore missed out on the financial reward). Therefore, we cannot determine whether rejection in general, or the perceived unfairness of the rejection caused the dishonest behavior. To test whether rejection in general, or the nature of a rejection causes people to behave dishonestly, we conducted a second experiment in which participants were rejected based on objective or subjective criteria.

## Study 2

### Methods and Materials

#### Participants and Design

One hundred and forty-four American MTurk workers participated in an online study on the effect of objective and subjective rejections on people’s mood and lying behavior. We selected a situation in which dishonesty can occur, and that can leave people with feelings of unfairness: filing an online insurance claim (Derrig, 2002; Topham, 2014). Participants filed a mock insurance claim form. General questions about the trip, such as departure airport and holiday destination, served as check questions. Seven MTurk workers failed to answer these questions correctly and were removed from the dataset, leaving 137 participants (75 female; age 19–72, *M* = 35.15, *SD* = 11.25). Participation took on average 14 min and participants received $1.00 for their time, consisting of a basic payment of $0.50 and a $0.50 bonus that every participant eventually received.

This study is a between-subjects design, measuring the effect of verdict (accept, objective reject vs. subjective reject) on falsifying information (falsified vs. not falsified information).

#### Procedure

Participants accessed our website via the MTurk platform and were told that this study was designed to test the accuracy and usability of an Automatic Validation Tool for online insurance claims. We asked participants to imagine that their backpack was stolen during a holiday trip in Europe, and they were asked to file a mock insurance claim form for this stolen backpack. To ensure consistency between sessions, participants were provided with a scenario describing how their backpack was stolen, and they received an overview of the main guidelines of ‘their’ travel insurance. This information was accessible through a pop-up menu whilst completing the insurance claim to ensure that participants would not make mistakes due to memory errors, rather than the deliberate falsification of information. To mimic a real-world situation, participants were promised a monetary reward (i.e., $0.50 bonus) upon claim acceptance. Both the insurance claim form and the policy guidelines were based on information provided by a large UK-based insurance company. The scenario, policy guidelines and claim form can be found at https://www.projects.science.uu.nl/lyingfeelsright/. Participants were also asked to complete two mood questionnaires, once before filing the claim and once after hearing the verdict on their claim, followed by the lie opportunity during which participants could falsify information on their insurance claim in order to get the claim accepted. Participants were led to believe this mood questionnaire to be part of testing the usability of the Automatic Validation Tool, while it actually was aimed at measuring whether people’s feelings were affected by a rejected claim.

The study started with demographic questions, followed by the presentation of the scenario and policy guidelines. Subsequently, participants filed an insurance claim based on the backpack scenario. After submitting their claim, participants saw the following message for several seconds: “Please wait a moment. We are now automatically checking the content of your insurance claim. Do not push the back button or refresh.” This message was followed by the verdict on their claim. After the verdict, people had the possibility to complain and/or make changes to their claim if they believed the Automatic Validation Tool had misunderstood what happened. Falsifying information (i.e., behaving dishonestly) was defined as submitting information that diverged from the information presented in the scenario, which could help participants get their claim accepted and win a reward.

Participants were randomly assigned to one of three Verdict conditions: accept, objective rejection and subjective rejection. In the accept condition, participants were told that, based on information they provided, their claim got accepted and that they would receive the bonus. In the objective rejection condition, participants were told that their claim was rejected because they had violated the maximum journey limit of 31 days. The scenario in all conditions was identical, except for the length of the journey in the objective rejection, which was 5 weeks instead of three, exceeding the insurance policy journey limit. We designed the violation of maximum journey length because it concerns a clearly-stated, common policy guideline, and one familiar to the general public. It can therefore be regarded as an objective rejection. Although this rejection was based on objective policy guidelines, participants still had the opportunity to cheat because they could change their departure or return date, or mention that they notified the insurance company of their extended journey beforehand.

The subjective rejection was based on the wide interpretation of the ambiguous statement that “people should take care to look after their personal possessions, in particular their valuables.” Participants were told that their claim was rejected because, based on the provided information, the conclusion had been drawn that they had been negligent in taking care of their possessions. There is no clear description of what behaviors do and do not count as negligence, making this a subjective rejection. Participants could falsify information by fabricating more convincing ways (i.e., not described in the provided scenario) in which they had taken care of their backpack. For example, one participant claimed that he had not left the backpack out of his sight, while it was clearly stated in the scenario that he/she only realized the backpack was missing when he/she was about to leave the restaurant. For each participant, a coder determined whether any information in the statement contradicted information provided in the scenario. For the objective rejection condition, this included mentioning incorrect dates and prior contact with the insurance company. For the subjective rejection, this included mentioning contact with the thief, keeping the backpack in sight at all times, and incorrect information about the location of their backpack. Other statements that participants made to increase the chances of getting their claim accepted, but which did not contradict information from the scenario such as claims of being an honest and careful person and portraying feelings of unfair treatment were not interpreted as false information. After the lie opportunity, participants were debriefed about the true purpose of the study, and all participants received the $0.50 bonus.

### Results and Discussion

**FIGURE 3:**
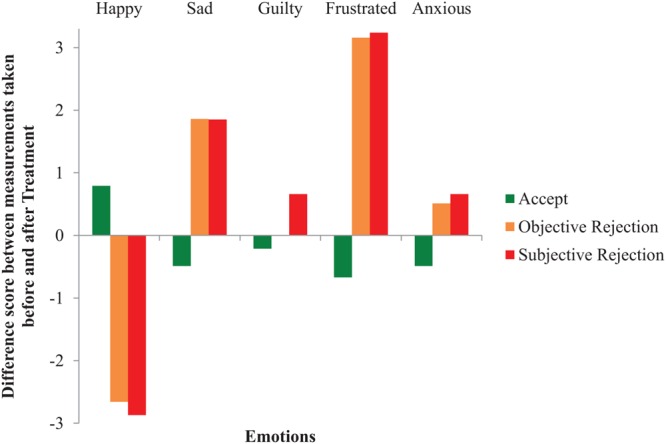
**The effect of Verdict on self-reported Mood (Study 2)**.

In line with Study 1, to measure participants’ emotions, five repeated-measures ANOVAs were performed with Verdict (i.e., accept, objective reject, and subjective reject) as the independent variable and the five self-reported Mood questions as the dependent variables. Results indicated with five interaction effects that participants’ mood was negatively affected by rejection in general, and that the nature of the rejection did not matter. Specifically, participants reported: (i) feeling less happy when getting rejected in general, compared to getting their claim accepted, *F*(2,134) = 73.13, *p* < 0.001, $\eta_{p}^{2}$ = 0.52; but, (ii) more sad when getting rejected in general, compared to getting their claim accepted, *F*(2,134) = 21.78, *p* < 0.001, $\eta_{p}^{2}$ = 0.25; (iii) more guilty when the rejection was subjective, compared to an objective rejection and acceptance, *F*(2,134) = 7.34, *p* = 0.001, $\eta_{p}^{2}$ = 0.10; (iv) more frustrated when rejected in general, compared to getting accepted, *F*(2,134) = 58.43, *p* < 0.001, $\eta_{p}^{2}$ = 0.47; and, (v) more anxious when rejected in general, compared to getting accepted, *F*(2,134) = 4.17, *p* = 0.018, $\eta_{p}^{2}$ = 0.02. See **Figure 3**, for a graphical interpretation of the results. These self-reported results indicate that, overall, participants’ mood was negatively affected by claim rejection, regardless of the nature of this rejection. Feelings of guilt were the only exception, as participants who were subjectively rejected felt guiltier after this than participants who were rejected based on measurable criteria or who were not rejected at all. A follow-up correlational analysis of Falsified information on self-reported Guilt in participants in the subjective rejection condition revealed that guilt was not induced by the dishonest behavior (i.e., falsifying information on the insurance claim form), *r* = 0.03, *n* = 47, *p* = 0.861.

**FIGURE 4:**
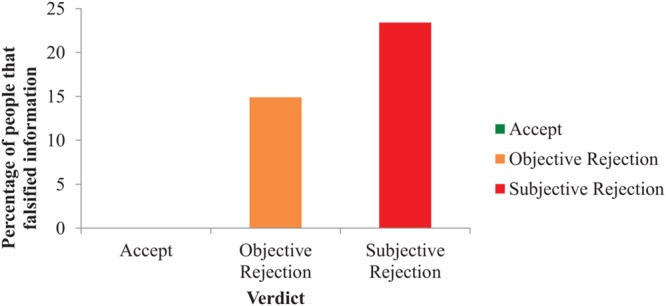
**The effect of Verdict on the percentage of participants that falsified information (i.e., lying; Study 2)**.

To measure whether rejection promoted dishonest behavior, a chi-square analysis of Verdict on the dependent variable Falsified information (i.e., yes or no) was performed. As demonstrated in **Figure 4**, people lie more after being rejected based on subjective criteria (23.4%) compared to rejection based on objective criteria (14.9%), and getting accepted (0%), *X*^2^(2) = 10.97, *n* = 137, *p* = 0.004, Φ = 0.28. To identify whether rejection in general caused this effect, or whether the nature of the rejection played a role as well, we ran an additional chi-square analysis in which we removed the accept condition. Results demonstrate that although participants falsified information more often when rejected subjectively by 8.5%, this difference between the two reject conditions was not significant, *X*^2^(1) = 1.09, *n* = 94, *p* = 0.294, Φ = 0.11. We next conducted a multiple mediation analysis following procedures by Preacher and Hayes (2008) to test whether self-reported mood (i.e., happiness, sadness, guilt, frustration, and anxiety) mediates the effect of rejection on dishonest behavior. We ran a bootstrapping analysis (5000 iterations) with the five mood variables simultaneously in the model and results indicated that only happiness [0.081 1.003] mediated the effect between rejection and dishonest behavior. The 95% bias corrected confidence intervals of sadness, guilt, frustration, and anxiety included zero, suggesting that these variables did not have a mediating effect.

In summary, rejection in general (i.e., regardless of the nature of this rejection), leads to negative emotions and more dishonest behavior. To which extent the nature of a rejection increases dishonest behavior, is a topic for further research. Participants falsified information more often and, independently, experienced more feelings of guilt when the rejection was based on subjective reasons. However, the chi-square analysis did not support this finding. Whether the difference in dishonesty between objective and subjective rejections was not significant due to a lack of power, or because dishonesty is predominantly driven by rejection rather than the nature of the rejection, cannot be determined based on the current data.

In the previous two studies, rejection always led to financial loss. Participants were also aware that they would profit financially from cheating (i.e., looking up the correct definition) and lying (i.e., falsifying information on an insurance claim to increase the chance of claim acceptance). Therefore, based on the results from Studies 1 and 2, we cannot disentangle whether people behaved dishonestly to compensate for their previous financial loss, or due to the rejection and negative emotions elicited by these rejections. We wondered, would this behavior change if there were no financial incentives associated with dishonesty? In other words, are people just trying to get back the money that was unfairly taken for them, or are they emotionally seeking revenge?

## Study 3

In the third and final experiment we tackled the confounding effect that dishonesty will often lead to financial gain (Greenberg, 1993; Houser et al., 2012; Kline et al., 2014); we removed the financial incentive to cheat in order to investigate whether financial incentives are the main motivator for people’s behavior when they have been rejected, based on either objective or subjective criteria.

### Methods and Materials

#### Participants and Design

One hundred and seventy-nine American MTurk workers participated in an online study on the effect of rejection and monetary rewards on people’s mood and lying behavior. Three participants failed to answer the check questions that were based on general scenario information correctly and were removed from the dataset, leaving 176 participants (110 female; age 18–68, *M* = 36.81, *SD* = 12.29). Participation took on average 14 min and participants received $1.00 for their time, consisting of a basic payment of $0.50 and a $0.50 bonus that every participant received.

This study is a 3 × 2 between-subjects design, measuring the effect of verdict (accept, objective reject vs. subjective reject) and bonus (bonus vs. bonus-after) on falsifying information (falsified vs. not falsified information).

#### Procedure

The procedure of Study 3 follows the procedure of Study 2 with one exception. Instead of telling all participants at the beginning of the experiment that they would receive a $0.50 bonus upon claim acceptance, half of the participants were not told about the bonus until the debriefing. In other words, participants in the bonus-after condition were not promised any financial bonus during the experiment and were only made aware of the existence of the bonus upon completion of the experiment. This way, we could measure the effect of a prospective bonus on mood and lying behavior. After the lie opportunity, participants were debriefed about the true purpose of the study, and all participants, including the participants in the bonus-after condition, received the $0.50 bonus at the end of the study.

### Results and Discussion

**FIGURE 5:**
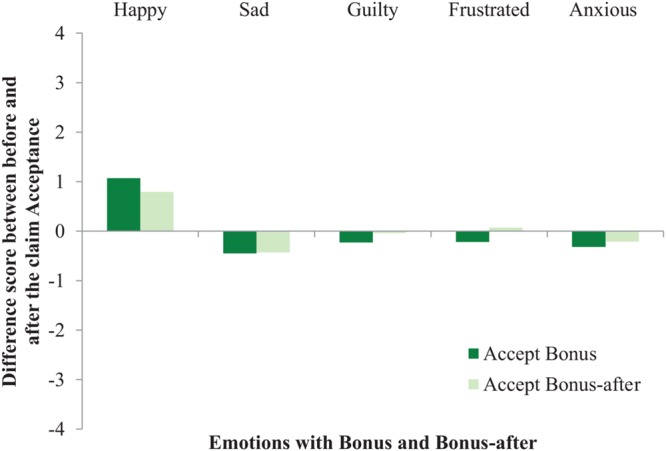
**The effect of Bonus on self-reported Mood in the Accept condition (Study 3)**.

**FIGURE 6:**
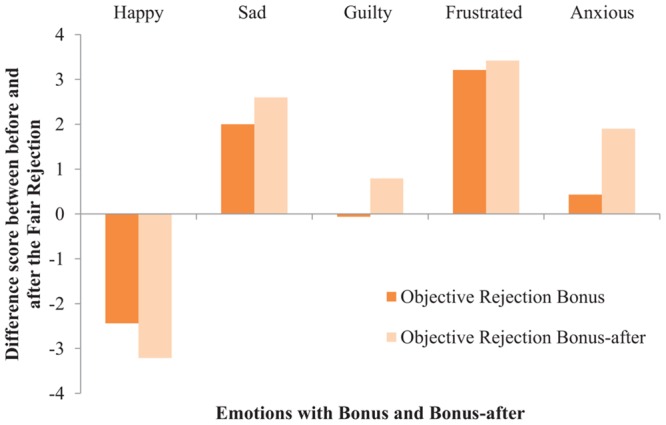
**The effect of Bonus on self-reported Mood in the Objective rejection condition (Study 3)**.

**FIGURE 7:**
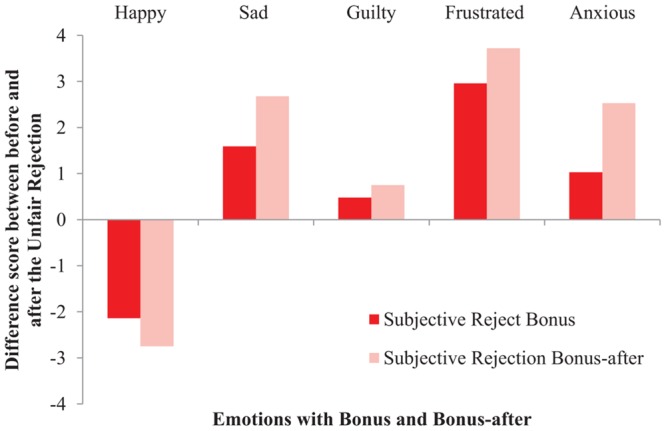
**The effect of Bonus on self-reported Mood in the Subjective Rejection condition (Study 3)**.

To measure if our Bonus manipulation affected participants’ emotions, five repeated-measures ANOVAs were conducted with Verdict (i.e., accept, objective reject, and subjective reject), and Bonus (i.e., bonus and bonus-after), as the independent variables and five self-reported Mood questions as the dependent variables. The mood results from Study 2 were replicated with five interaction effects, indicating that participants’ mood was negatively affected by getting a claim rejected in general, regardless of the nature of a rejection. Specifically, participants reported: (i) feeling less happy when getting rejected in general, compared to accepted, *F*(2,170) = 84.57, *p* < 0.001, $\eta_{p}^{2}$ = 0.50; but, (ii) more sad when getting their claim rejected in general, compared to accepted, *F*(2,170) = 33.61, *p* < 0.001, $\eta_{p}^{2}$ = 0.28; (iii) more guilty when rejected in general, *F*(2,170) = 4.89, *p* = 0.009, $\eta_{p}^{2}$ = 0.05; (iv) more frustrated when rejected in general, *F*(2,170) = 49.40, *p* < 0.001, $\eta_{p}^{2}$ = 0.37; and, (v) more anxious when rejected in general, *F*(2,170) = 20.48, *p* < 0.001, $\eta_{p}^{2}$ = 0.19. See **Figures 5**–**7** for graphical interpretations of these results. Importantly, in addition to Verdict, Bonus also affected people’s mood. Participants reported feeling less happy after hearing the verdict about their claim when they were not aware of the bonus (*M* = 3.37, *SD* = 2.22), compared to situations in which participants received a bonus upon claim acceptance (*M* = 3.78, *SD* = 2.20), *F*(1,170) = 4.57, *p* = 0.034, $\eta_{p}^{2}$ = 0.03. Participants also reported feeling more guilty after hearing the verdict in the bonus-after condition (*M* = 2.00, *SD* = 1.48), compared to scenarios where participants received a bonus, (*M* = 1.62, *SD* = 1.15), *F*(1,170) = 4.85, *p* = 0.029, $\eta_{p}^{2}$ = 0.03. Lastly, participants reported feeling more anxious after hearing the verdict in the bonus-after condition (*M* = 3.23, *SD* = 2.13), compared to the bonus condition (*M* = 2.49, *SD* = 1.99), *F*(1,170) = 14.52, *p* < 0.001, $\eta_{p}^{2}$ = 0.08. These self-reported mood results indicate that, overall, participants’ mood was negatively affected when their claim was rejected, regardless of the nature of this rejection. These results also indicate that people felt emotions more strongly when there was seemingly no money involved (i.e., decreased happiness, increased guilt and anxiety).

**FIGURE 8:**
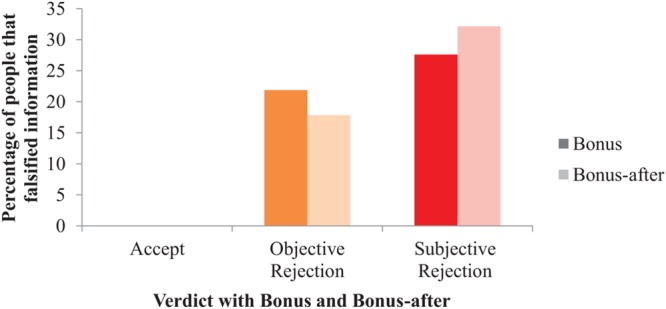
**The effect of Verdict and Bonus on the percentage of participants that falsified information (i.e., lying; Study 3)**.

To measure whether a monetary bonus not only affected people’s mood, but also their tendency to behave dishonestly, a loglinear analysis of Verdict (i.e., accept, objective reject, and subjective reject), and Bonus (i.e., bonus and bonus-after) on Falsified information (i.e., yes or no) was performed. In other words, we analyzed if people’s tendency to lie was dependent on monetary rewards and the nature of their claim rejection. In line with standard practice, 0.5 was added to all cells to avoid performing calculations with empty cells. The loglinear regression revealed that the highest order three-way model did not retain all effects. Instead, the best fit was a second-order model, *X*^2^(0) = 0, *p* = 1, including a two-way interaction effect between Verdict and Falsified information, *X*^2^(5) = 28.08, *n* = 176, *p* < 0.001. A separate chi-square analysis of Verdict on Falsified information demonstrated a significant difference between the conditions unfairly rejected (29.8%), fairly rejected (20.0%), and accepted (0%), *X*^2^(2) = 19.56, *n* = 176, *p* < 0.001, Φ = 0.33, see **Figure 8**. Although participants in the subjective reject condition cheated almost 10% more (and relatively 49% more) than participants in the objective reject condition, this effect did not differ significantly when tested without the accept condition, *X*^2^(1) = 1.51, *n* = 117, *p* = 0.219, Φ = 0.11. The influence of Bonus on Falsified Information was not further tested because these factors were not included in the best fitting loglinear regression model. We next conducted two multiple mediation analyses to test whether self-reported mood (i.e., happiness, sadness, guilt, frustration, and anxiety) mediates the effect of rejection on dishonest behavior. Because the presence or seemingly absence of a bonus influenced how rejection affected people’s mood, we split the file up based on Bonus condition and ran two separate analyses. We ran two bootstrapping analyses, one for the bonus and one for the bonus-after condition (5000 iterations each) with the five mood variables simultaneously in the model. Results indicated that none of the emotions mediated the effect of rejection on subsequent dishonest behavior.

In conclusion, the mood results demonstrate that people experienced the negative emotions associated with rejection more strongly when there was no financial reward involved, although these strong emotions did not subsequently increase dishonest behavior. Dishonesty was also not affected by the presence of a financial reward, or by the nature of the rejection. Instead, rejections in general fueled dishonest behavior. So the absence of money made people care more, but it did not spark dishonesty: being rejected did.

## General Discussion

In previous research, dishonesty was often quantified as cheating (Houser et al., 2012) or stealing (Greenberg, 1993). Because dishonesty encompasses more behaviors than just stealing and cheating, dishonest behavior in this paper was not only quantified as cheating (i.e., on a test; Study 1), but also as falsifying information (i.e., on a mock insurance claim; Studies 2 and 3). These types of dishonest behaviors are, for example, relevant in the applied context of insurance claims. Because insurance claims are nowadays often filed online, the studies in this paper were conducted using the online platform Mechanical Turk.

In three studies, we have investigated whether the rejection of one’s efforts elicits dishonest behavior in an online setting. In Study 1, we pretended to run a language proficiency study and rejected the efforts of half of the participants by providing them with negative feedback about their performance. People who were rejected subsequently engaged more often in unethical behavior when they got the chance than people who did not previously got rejected. Although this study provided an interesting insight in online dishonest behavior, we discovered two possibly confounding factors. First, because participants in the reject condition were rejected unfairly (i.e., although they had answered all questions correctly, they were told they made a mistake), we could not determine whether the rejection in itself, or the nature of the rejection caused the rise in dishonest behavior. Second, the rejection resulted in financial loss, which meant we could not determine whether the rejection, or the financial loss associated with the rejection caused people to behave dishonestly. In Study 2, we tested whether the nature of the rejection, operationalized as rejection based on objective or subjective criteria, affected dishonest behavior. In Study 3, we investigated the role of financial rewards in the motivations for dishonest behavior. These two studies were conducted in an online insurance claim environment for its relevance in our current society. Results indicated that across experiments, the rejection, rather than the nature of a rejection, promoted dishonest behavior. Although participants cheated approximately 10% more (a relative difference of 49%) after being rejected for subjective reasons compared to objective criteria, this difference was not statistically significant. Based on the current data we cannot determine whether the nature of a rejection simply does not affect dishonest behavior, or whether the lack of effect was due to a lack in power. Other papers investigating the effect of treatment on dishonesty experienced similar power problems. For example, the difference in dishonest behavior between the fair and unfair condition in Houser et al.’s (2012) study was only marginally significant with a sample size of 500+. This suggests that the effect size of fairness on dishonesty may be relatively small. Regardless, decreasing dishonest behavior in the context of insurance claims with a few percent can still lead to large financial benefits, making this topic worth exploring.

When removing the financial rewards associated with dishonest behavior, participants still made an effort to falsify information, suggesting that dishonesty is not just caused by an attempt to get restorative justice for missing out. Although having a financial reward associated with accepted claims – as is common in real-life insurance claims – affected people’s feelings, it did not affect dishonest behavior. These results support previous theory (Ariely, 2012) and experimental results (Mazar et al., 2008) on the irrationality of dishonesty, which demonstrates that people do not base their decision to behave dishonestly on a rational cost-benefit analysis. We tested this by adding the bonus-after condition in Study 3, so in the perception of the participants we removed the (financial) benefits of acting dishonestly, while the costs in terms of effort did not change. If people were rational economic actors, the seemingly absence of financial rewards would have stopped them from cheating. However, dishonest behavior was not affected. Rather, when there are no financial gains in prospect, emotional involvement was larger. It is as if playing for honor is more important than playing for money. When unaware of any prospective reward, participants indicated feeling less happy, and more guilty and anxious after hearing the verdict about their claim than people who had hoped for financial benefits. Importantly, although the financial benefits in Study 3 were small, they still elicited emotional and behavioral changes. Larger incentives would not necessarily have increased this effect, just as Ariely (2012) demonstrated that increasing the financial incentive did not lead to increased cheating. Moreover, Ruedy et al. (2013) replaced the financial incentive with a more personal incentive to cheat by linking success on the test to intelligence and professional success in life and found that people cheated significantly more when their self-esteem was at stake.

A theory that may help explain these irrational dishonesty results is ‘ethical dissonance’ (Barkan et al., 2015), a theory that is related to the general ‘cognitive dissonance’ theory by Festinger (1957) in which internal consistency is threatened by two or more conflicting beliefs and ideas. Specifically, in our insurance claim studies, ethical dissonance may have occurred when people tried to justify to themselves why they spent time and effort (i.e., adding feedback and falsifying information on the claim form) without any potential benefits (i.e., no monetary bonus). The friction caused by these conflicting beliefs may then be solved by stating that they made this effort because they care (i.e., higher emotional involvement). The ethical dissonance theory (Barkan et al., 2015) describes how people feel torn between wanting to be a good person (Aronson, 1969; Josephson Institute of Ethics, 2012), and wanting the benefits of behaving dishonestly (Mazar et al., 2008). Although we did not directly ask participants how they felt about themselves in order to avoid priming (dis)honest behavior (Mazar et al., 2008; Shu et al., 2012), the implemented mood questionnaires can be used as an indication of their mental states. In previous research, dishonest behavior has been linked both to eliciting negative emotions such as guilt (Massi, 2005) and to eliciting positive affect (Ruedy et al., 2013). Here, the mood results from the first study showed that cheating did not affect people’s emotions, suggesting that our participants may have been effective at justifying their dishonest behavior. This is key, because dishonesty can be a slippery slope (Lerman, 2002; Ariely, 2012). If people can behave dishonestly and still feel good or even better (Ruedy et al., 2013) about themselves, they might be more likely to behave dishonestly again in the future.

The chosen experimental designs have several benefits including the ability to test multiple types of dishonest behavior. This allowed us to investigate aspects of dishonesty that go beyond simple tasks such as reporting the outcome of a coin toss (Houser et al., 2012), and analyze dishonest behavior in more realistic settings. However, when participants complete a study on their own computers, this typically reduces the amount of experimental control. Specifically in Study 1, we could not distinguish between participants who quit in an attempt to cheat, and those who quit for other reasons such as frustration or lack of trust in the system. In Studies 2 and 3, participants might have reported more negative emotions, not as the sole result of the feedback decision, but caused by a discrepancy between behavior dictated by their assignment, and the behavior that would have led to the highest gain. More specifically, when participants in the objective rejection condition filled out the travel dates conscientiously, their claim would get rejected. While the online insurance fraud scenario provides both realism and a structured experimental testing mechanism for dishonest behavior, the scenarios and instructions may have posed conflicting incentives for the participants. In addition, much dishonesty research shows that people usually cheat and lie a little (Ariely, 2012; Houser et al., 2012). The complete absence of falsifying information in the accept conditions of Studies 2 and 3 is likely to have been caused by the choice of experimental design because claim acceptance, and therefore pay-off, was quantified as a binary decision. In other words, an accepted claim led to the highest achievable monetary reward and therefore did not require participants to falsify additional details, whilst in real life people could still inflate their claim a little, and thus receive more money.

The consistency of our dishonesty findings across three studies and two research designs strengthens our belief that people behave more dishonestly after rejection, specifically in an online environment. In an applied setting, this would imply that firms should try to minimize the amount of rejected claims, for example through heightened transparency and clearer communication of acceptance guidelines. When it is upfront clear whether a claim is likely to be accepted or not, people may submit less claims that clearly violate policy guidelines, leading to a reduction in rejection and thus subsequent dishonest behavior. Despite the lack of a statistically significant difference, likelihood ratios indicated that the nature of a rejection may contribute to the elicitation of dishonest behavior as well. Because even a small decrease in fraudulent insurance claims can lead to a large savings, stating the rejection policy more clearly could not only reduce the amount of rejected claims, it may also further reduce dishonest behavior when people feel that they were rejected on objective grounds.

Although rejection did cause people to feel more negative (i.e., less happy, more sad, frustrated, and anxious), this emotional response did not have a strong effect on dishonest behavior. The mediation analyses of Studies 2 and 3 indicated that the majority of emotions did not affect dishonesty as a mediator (i.e., indirectly). Only the reduction in happiness in Study 2 mediated the effect between rejection and dishonesty. Anxiety, a previously demonstrated mediator in the context of social rejection (Kouchaki and Wareham, 2015), did not have a similar effect in our studies. There are several hypothetical explanations for the discrepancy between our findings and the existing literature on this topic. First, social rejections may elicit a different response than rejected efforts. The relationship between social rejection and (social) anxiety is well explored and lies at the core of human functioning (Baumeister and Tice, 1990; Leary, 1990). The rejection of one’s efforts may play a less central role and therefore have a weaker corresponding anxiety effect. A second possible explanation could be that people behave differently online, compared to face-to-face situations. The majority of research on factors that affect dishonest behavior has been conducted in lab experiments, but the few studies that have investigated dishonesty in an online context have demonstrated that the extent to which people behave dishonestly is affected by the modality of their interaction. For example, Zimbler and Feldman (2011) found that people tend behave more dishonestly when interacting online.

A factor that may have mediated the effect between rejection and dishonesty, but which we did not explicitly test, is fairness. In Studies 2 and 3, we differentiated between rejections based on objective and subjective grounds. Especially the rejections on subjective grounds may have elicited feelings of unfairness. Previous research has demonstrated that fairness can induce dishonest feelings (e.g., satisfaction levels; Hegtvedt and Killian, 1999), plans (e.g., hypothetical dishonest behavior; Schweitzer and Gibson, 2008), and even behavior (e.g., selfish behavior, Kline et al., 2014; cheating, Houser et al., 2012; and stealing money, Greenberg, 1993). Fairness has also proven be to an influential factor when it comes to online behavior, as the fairness of a request was the best predictor of honest behavior in a personal information disclosure study (Malheiros et al., 2013). Whether violations of fairness mediate the effect between rejection and dishonesty, will need to be explored in future research. If fairness turns out to be influential, firms can further experiment with attempting to adapt their customers’ fairness perceptions. The fairness of a situation is often ambiguous (Van den Bos et al., 1997), and fairness perceptions can be influenced (Bies and Shapiro, 1987; Egelman et al., 2010). In other words, violations of fairness principles may be used to justify dishonest behavior that is ambiguous, a factor that has repeatedly been shown to justify dishonesty (Schweitzer and Hsee, 2002; Shalvi et al., 2015). Therefore, investigating what factors determine whether customers interpret a situation as fair will allow firms to promote honest behavior by tipping the conflict between wanting to be a good person and the benefits of dishonesty in the honest direction (Bies and Shapiro, 1987). Transparency may be the way for firms to see to it that their customers do not feel that lying is the right thing to do, potentially reducing the cost of opportunistic fraud.

## Author Contributions

All authors helped design the experiments and commented on the paper. SV collected the data and wrote the initial draft. RP created the framework for the online data collection. All co-authors revised the paper for resubmission.

## Conflict of Interest Statement

The authors declare that the research was conducted in the absence of any commercial or financial relationships that could be construed as a potential conflict of interest.
